# Effect of bisphenol A on alterations of *ICAM-1* and *HLA-G* genes expression and DNA methylation profiles in cumulus cells of infertile women with poor response to ovarian stimulation

**DOI:** 10.1038/s41598-021-87175-1

**Published:** 2021-05-05

**Authors:** Somayeh Aftabsavad, Zahra Noormohammadi, Ashraf Moini, Morteza Karimipoor

**Affiliations:** 1 Department of Biology, Science and Research Branch, Islamic Azad University, Tehran, Iran; 2 Department of Endocrinology and Female Infertility, Reproductive Biomedicine Research Center, Royan Institute for Reproductive Biomedicine, ACECR, Tehran, Iran; 3 Breast Disease Research Center (BDRC), Tehran University Of Medical Science, Tehran, Iran; 4 Department of Obstetrics and Gynecology, Arash Women’s Hospital, Tehran University of Medical Sciences, Tehran, Iran; 5 Department of Molecular Medicine, Biotechnology Research Center, Pasteur Institute of Iran, Tehran, Iran

**Keywords:** Genetics, Molecular biology, Biomarkers, Molecular medicine

## Abstract

This study aimed to investigate the relationship between follicular fluid Bisphenol A (BPA) concentrations with alterations of ICAM-1 and HLA-G genes and proteins expression as well as methylation profiles in the cumulus cells of poor ovarian response (POR) women based on their healthy lifestyle habit. Eighty women under the age of 35 were divided into two groups: 1—POR without using plastic containers (n = 40) and 2—POR with using plastic containers (n = 40). The ICAM-1 and HLA-G genes and protein expressions were examined by the quantitative PCR and western blotting technique. The methylation pattern was investigated by the methylation-specific PCR. Total BPA in follicular fluid was measured with high-performance liquid chromatography technique and the detection limit was 1.14 ng/ml. ICAM-1 and HLA-G genes were differentially expressed between the two groups studied. ICAM-1, HLA-G genes, and protein expressions in group 1 were up-regulated compared to the second group (P < 0.05). While DNA methylation status in group 1 were decreased compared to the other group (P < 0.05). The concentration of BPA in the follicular fluid of group 1 was lower compared to the second group (P < 0.05). The oocyte quality and clinical pregnancy ratio showed significantly higher in group 1 than in the other ones (P < 0.05). The alteration of ICAM-1 and HLA-G gene expressions in POR women is probably related to BPA concentration. As a result Lifestyle habits may also affect the methylation pattern and protein levels in the cumulus cells of POR women. Additionally, lifestyle habits may be considered as a marker for ovulation, oocyte maturation, preimplantation, and clinical pregnancy process.

## Introduction

The term poor response to ovarian stimulation (POR) typically refers to woman with a reduced ovarian reserve or poor ovarian response to exogenous gonadotropin stimulation^1^. In fact, the POR or the cycle without ovulation is a cycle in which the ovary is unable to release the oocyte. As a result, ovulation does not occur. Despite the efforts to optimize the definition of POR, unfortunately there is still limited knowledge about the POR pathophysiology, and the etiology hinders the practical solutions to manage the condition^2^. Indeed, the diagnosis of anovulation is not easy. Contrary to popular belief, women with anovulation have more or less a normal menstrual cycle and usually for the first time, they find their own problem when they want to become pregnant^3^. Recently, POSEIDON criteria have been used to diagnose POR, including age, Anti Müllerian Hormone (AMH) dose, Antra Follicle Count (AFC), and oocyte number^4^. The consequential complications of POR are intrinsic ovarian resistance to gonadotropin stimulation, reduced ovarian reserve, ovulation and oocyte maturation disorder^5^. Reduced ovarian reserve is defined as the impaired fertility ability leading to infertility. In fact, excessive use of plastics objects in the human life has been proposed as one of the causes of creating POR in women^6^. Indeed, excessive use of plastics to store hot food and drinks can cause various diseases. Polycarbonate is a type of plastic containing BisphenolA (BPA)^7^. Over time, when plastic is heated or exposed to acidic or alkaline substances, it breaks down and BPA attaches to food and drink, so that it can enter the body^8^. BPA, by mimicking sex hormones, can disrupt the endocrine system, hormones, hormonal signaling pathways and gene expression^9^. BPA even in small amounts may interfere with the ovulation, ovarian response, oocyte maturation, pregnancy and genes function^10^. In fact, lifestyle habit plays a key role in regulating ovarian response, oocyte maturation and genes expression. Gene’s expression alteration in POR patients is a condition damaging oocyte developmental, resulting in reduced rates of fertilization, embryonic development, and implantation^11^. The role of gene’s expression alteration in the pathophysiology and etiology of POR is uncertain, but studies support the hypothesis that nutrition and lifestyle habit play a crucial role in regulating the response of intrinsic ovarian resistance to gonadotropin stimulation, reduced ovarian reserve, oocyte maturation, implantation and gene expression^12^.

ICAM-1, known as CD54, plays a pivotal role during oocyte maturation, and it is a protein encoded by the *ICAM1* gene. The responsibility of *ICAM-1* is encoding a cell surface glycoprotein usually expressed on endothelial cells and cells of the immune system. As mentioned, ICAM-1 plays a central role in the ovulation process^13^. Indeed, the main components of this ovulation process are the disruption of the extracellular matrix (ECM) at the follicular peak and the change in follicular vessels. In these events, ICAM-1 participates by secreting proteases and active inhibitors. Indeed, biochemical markers of the oocyte maturation and ovulation are highly important. Upregulation of *ICAM-1* in immature oocytes as well as downregulation of *ICAM-1* in mature oocytes has been observed^14^. Human leukocyte antigen-G (HLA-G) is regarded to play a vital role in oocyte maturation and implantation of embryos. HLA-G can be effective in controlling trophoblast invasion and maintaining a local immunosuppressive state. The expression and distribution of *HLA-G* is on human spermatogenic cells, primary and secondary oocytes, and preimplantation embryos. Furthermore, the *HLA-G* production is related to the good quality of cumulus cells (CCs) in oocytes^11^.

In the present study, we aimed to assess the relationship between follicular fluid BPA concentrations with genes expression, proteins level and methylation status of *ICAM-1* and *HLA-G* in the cumulus cells of infertile POR patients based on their lifestyle habit following ovarian stimulation with a gonadotropin releasing hormone (GnRH) antagonist protocol.

## Materials and methods

### Patient selection

In the present study, eighty women under the age of 35 and BMI 18–25 kg/m^2^, who participated in an intracytoplasmic sperm injection (ICSI) program, were selected. On the day of ovarian puncture, follicular fluid sampleof each woman was collected in a special tube (without using BPA compound, it is called healthy lifestyle habit here) and then stored at − 70 °C until analysis. The patients who were diagnosed by the Poseidon group1 subgroup 1b that included; [patients < 35 years old with adequate ovarian reserve parameters (AFC > 5; AMH > 1.2 ng/ml) and with an unexpected poor or suboptimal ovarian response, and 4–9 oocytes retrieved] (4). The patients were divided into two groups: without a healthy lifestyle habit (control group contain, n = 40, infertile women with poor response (POR) to ovarian stimulation history), and with a healthy lifestyle habit (case group contain, n = 40, infertile women with poor response (POR) to ovarian stimulation history). The healthy lifestyle habit group included women who used little plastic containers for hot food, hot drink and foodstuffs store. However, the group without a healthy lifestyle habit included women who used excessive plastic containers to store hot food, hot drink and foodstuffs. Information on a healthy lifestyle habit was obtained using a validated food frequency questionnaire. Our questionnaire includes precise questions such as; how much they use plastic containers for the hot food, hot drink, and foodstuffs store in their daily life. The main exclusion criteria were women affected by endometriosis, and women with a history of uterus and ovaries operation. Furthermore, in this study, at the request of the patients, the treatment IVF/ICSIcycle was canceled, while they had a low number of follicles or lacked growth of follicles. The Ethics Committee at Royan Institute, Iran approved this prospective case–control study (No. IR.ACECR.ROYAN.REC.1394.150). All participants gave informed consent prior to inclusion in the study. We ensured the confidentiality of the patients’ identities by data anonymization during analysis. This research did not affect the treatment of patients.

### Flexible antagonist stimulation protocol

In this study, controlled of ovarian stimulation (COS) was controlled from the third day of the cycle. The patients received regular, daily subcutaneous (SC) injections of recombinant follicle stimulating hormone (rFSH, Gonal-F, Serono, Switzerland). The first dose of rFSH for each patient was started according to sonographic monitoring, and AFC, estradiol (E2) level, and AMH for each patient were evaluated. In the stage of growing follicles > 12 mm, the patients received SC injections of a GnRH antagonist, cetrorelix (Cetrotide, Merck Serono, Germany). The protocol consisted of daily Cetrotide SC injections until the criteria for human chorionic gonadotropin (hCG) administration were met. When more than 3 follicles reached diameters of at least 18 mm and E2 levels of 1000–4000 pg/mL When at least three follicles reached diameters of ≥ 18 mm as well as E2 levels of 1000–4000 pg/mL, each patient received an intramuscular (IM) injection of 10,000 IU of hCG (Pregnyl, Organon, Netherlands) or SC injection of 250 μg Ovidrel (Merck Serono, Germany).

### Isolation of cumulus cells

Following follicular puncture, the cumulus-oocyte complexes (COCs) were collected and washed at least 3–5 times in G-IVFTM medium (Vitrolife, Sweden) to remove blood and excess cells. Immediately after washing, the COCs were moved to a CO_2_ incubator at 37 °C for 2 h in G-IVFTM medium. Cumulus cells were denuded from COCs by 80 IU of hyaluronidase, (Sigma, USA). Cumulus cells from each mature oocyte (100 in POR with healthy life style versus 100 in POR without healthy life style) were collected in individually tubes. After oocyte denudation, a pellet of cumulus cells was washed with phosphate-buffered saline (PBS), and RNA stabilizer reagent buffer was added for further analyses. Cumulus cells were immediately transferred to liquid nitrogen for snap freeze, and then they were stored at – 80 °C. The cumulus cells were denuded from metaphase II gametes (MII). In the IVF laboratory, MII gametes were fertilized by the ICSI process within 10 min after denudation, and they were incubated until the embryo transfer was processed. Fertilization was evaluated (16–20 h after ICSI). According to our IVF laboratory standards process, embryos were graded by embryologist specialists at the pronuclear (16–20 h) and cleavage stages (48–72 h). Then, the embryologist selected 1 or 2 embryos for transfer. For successful embryo transfer, they considered the embryo grades, age of the patients, and previous ART cycles. The quality of the embryos at the cleavage stage was classified based on these criteria: [The excellent quality of embryo should be (≥ 4 cells or ≥ 8 cellsand < 10% fragmentation), the good quality of embryo should be (≥ 4 cells or ≥ 8 cells and 10–20% fragmentation), and the poor quality of embryo should be (< 4 cells or < 8 cells and > 20% fragmentation)]^15^.

### Follicular fluids bisphenol A analysis

The samples were analyzed at Analytical Chemistry Laboratory of Sharif University. Total BPA (conjugated and free) in follicular fluid was measured using high-performance liquid chromatography (HPLC). Briefly, at the first, in the glass tubes reaction mixtures contains of phosphorous acid buffer, β-glucuronidase (Sigma, USA), and sample aliquots was made. Afterwards, hydrolyzations at 37 °C were done, and then extracted twice with ether (HPLC grade, Merck, Germany). With the help of nitrogen gas flow, the supernatants were collected and evaporated. The remaining material was dissolved in 60% acetonitrile (HPLC grade, Merck, Germany) and HPLC was used for analysis: A KNAUER liquid chromatograph (KNAUER, Germany) with a RF-20A bulk fluorescence detector with an excitation wavelength of 275 nm and an emission wavelength of 300 nm was used. Chrom Gate software version 3.3 (KNAUER, Germany) was used for data processing. In the following, we used 5 μM Columns (Chromolith Performance RP-18e, USA), and columns 100 × 4.6 mm for liquid chromatography, and 20 μl injection loop, mobile phase A and B, acetonitrile/water (40:60, v/v), equivalent grade; and flow: 1.0 mL/min. HPLC water was used from Millipore Super-Q Plus Water Purification System (Bedford, MA, USA). In this experiment, for the calculating of limit of detection (LOD) we used of the Environmental Protection Agency method (2004). The LODs of BPA in follicular fluid were 1.14 ng/ml.

### qPCR

Total RNA was extracted by a Trizol (TRI; Sigma-Aldrich, USA) and treated with RNase-free DNaseI according to the manufacturer’s instructions. RNA concentration and purity were quantified using a Nanodrop2000 Spectrophotometer (Thermo, USA). Reverse transcription was performed at 25 ºC for 10 min, 42 ºC for 1 h and then 70 ºC for 10 min. For this reaction, cDNA was synthesized using the Revert AidTM H-Minus First Strand cDNA synthesis (Cat NO: K1631, Fermentas, Germany) by the random Hexa nucleotides primer (0.2 µg/µl) in a solution (20 µl) containing 4 µl 5× reaction buffer, 1 µl Ribonuclease inhibitor (20 U/µl), 2 µl dNTP mix (10 mM) and RNase-DNase free water.

*ICAM-1* and *HLA-G* were chosen as target genes, and 18srRNA was utilized normalizae each sample. Primers were designed by the Primer Express 3.0 software for the real-time PCR. Table 1 presents the primer sequences. The real-time PCR was performed in a StepOnePlus instrument (Applied Biosystems, USA). The reaction contained 2 µl cDNA, 10 µl of Power SYBR Green Master mix (Cat NO: RR420A, TAKARA, Japan) and 1 µl (of 500 nM primer) of forward and reverse primers. The reactions were performed in duplicate. The thermal cycling condition for the amplification was 95 °C for 10 min, followed by 40 cycles of 95 °C for 15 s and 60° for 10 min. The Ct data were determined using default threshold settings. The 2^−ΔΔCT^ method was used to analyze the relative expression level of target genes. Melt curve analysis was performed to determine the specificity of the real-time PCR assay. A t-test was used to investigate whether the differences between *ICAM-1* and *HLA-G* genes expression were calculated by the 2^−ΔΔCT^ method.

**Table 1:** Real time gene expression and MSP primers sequences.

Primers for real time gene expression	Sequences	Product size (bp)	
F-*ICAM*-1	5′-GCAATGTGCAAGAAGATAGCCA-3′	105	
R-*ICAM*-1	5′-GGGCAAGACCTCAGGTCATGT-3′		
F-*HLA*-*G*	5′-AGCTGTGGTGGTGCCTTC-3′	106	
R-*HLA*-*G*	5′-GGGCAGGGAAGACTGCTT-3′		
F-*18srRNA*	5′- GTAACCCGTTGAACCCCATT-3′	151	
R-*18srRNA*	5′- CCATCCAATCGGTAGTAGCG-3′		

Primers for MSP	Sequences	Product size	CpG Island Info
F-*ICAM1*-*M*	CGCGATTTTTTTGGTTTTTC	121	chr19:10269612–10271289
R-*ICAM1*-*M*	TATTTACTTAACCACCGCCTATACG		
F-*ICAM1*-*UM*	GTTGTGTGATTTTTTTGGTTTTTT	121	
R-*ICAM1*-*UM*	TTTACTTAACCACCACCTATACATA		
F-*HLA G*-*M*	CGTAGGTATATTGTTTATATTCGCG	120	chr6:29827777–29828817
R-*HLAG*-*M*	TACCTAAAAAAACCCCAAAACG		
F-*HLAG*-*UM*	TGTAGGTATATTGTTTATATTTGTGG	120	
R-*HLAG*-*UM*	CTACCTAAAAAAACCCCAAAACAC		

### Western blot analysis

**Figure 1:**
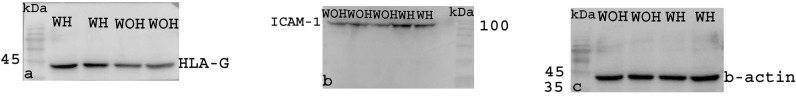
Comparison of (**a**) HLA-G (43 kDa), (**b**) ICAM-1 (100 kDa), and (**c**) β actin protein as control. Protein levels in western blot analysis were examined between POR patients without healthy lifestyle habit and with healthy lifestyle habit. In the (**a**) and (**b**) pictures, WH lanes belong to the POR patients with healthy lifestyle habit and WOH lanes belong to the POR patients without healthy lifestyle habit (Supplementary Information).

The total protein was extracted from equal amounts of cumulus cells in all samples using lysis buffer (7 M urea, 2 Mthiourea, 4% CHAPS [w/v], 75 mM DTT, 1% ampholyte [w/v], and 40 mMTris‐HCl), and then the protein concentration was evaluated by bicinchoninic acid assay (Thermo Scientific, Rockford, Illinois, USA). Additionally, 40 μg of protein for each sample was used onto a discontinuous 12% SDS‐polyacrylamide gel and exposed to vertical electrophoresis. Afterward, the proteins were transferred to poly vinylidenedi fluoride membranes (Bio‐Rad, Hercules, USA). Subsequently, with 3% bovine serum albumin (Sigma‐Aldrich, USA) as well as 1% of nonfat dry milk (Amersham, GE Healthcare Life Sciences, Little Chalfont, UK), the membranes blocked process was performed. The time required for membranes blocked process is 2 h at RT. In this stage, the membranes were incubated for overnight at 4 °C with primary antibodies against human ICAM1 (G-5) (1:1000, Cat. No.: SC‐8439; Santa Cruz, Madeira, Spain), HLA-G (4H84) (1:1000, Cat.No.: sc-21799; Santa Cruz, Madeira, Spain) and β‐actin (1:1000, Cat. No.: A2228; Sigma‐Aldrich, USA). In the next stage, the membranes secondary antibodies were used. Indeed, for 1.5 h in RT, the membranes were exposed to horseradish peroxidase‐conjugated secondary antibodies. The identification of ICAM1, HLA-G and β‐actin proteins was gained by goat antirabbit IgG (1:5000, Cat. No.: ab6112; Abcam, Cambridge, UK), and antimouse IgG (1:10,000, Cat.No.: 7076; Cell Signaling Technology, Danvers, USA) immunoglobulins, respectively (Fig. 1). Chemiluminescence detection system (UVITEC, UK) was used for protein visualization. The ImageJ software version 1.50i (US National Institutes of Health, Bethesda) was used to quantify protein bands intensity on the PVDF paper^16,17^. The changes in ICAM-1 and HLA-G level were normalized against β‐actin as a housekeeping protein and then calculated regarding infertile women with a poor response to ovarian stimulation without a healthy lifestyle habit.

### Methylation-specific polymerase chain reaction (MSP)

Genomic DNA was extracted from COCs by the QIAamp DNA Micro Kit (Cat NO: 56304, QIAGEN, Netherlands) according to the manufacturer’s instructions. DNA quantity and quality were measured using a Nanodrop 2000 Spectrophotometer (Thermo, USA). Genomic DNA was treated by bisulfite using the EZ DNA Methylation-Gold (Zymo Research, Germany) according to the manufacturer’s instructions. After conversion, DNA was eluted in buffer (Qiagen, Germany) to a final concentration of 30 ng/μl.

**Figure 2:**
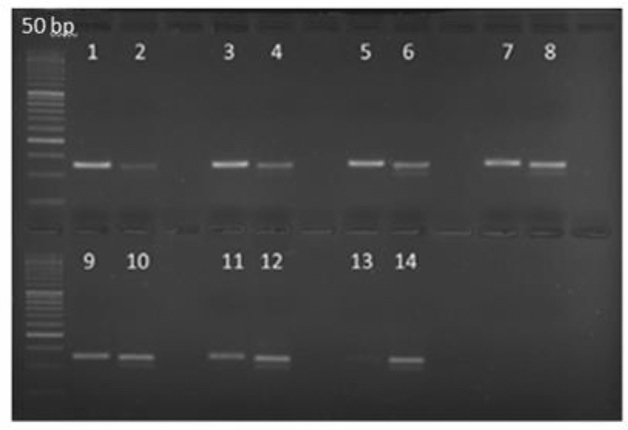
Methylation profiling of HLA-G and *ICAM-1* by methylation-specific PCR (MSP). Results of MSP on HLA-G and *ICAM-1* promoters from gDNA isolated from with healthy lifestyle habit and without healthy lifestyle habit of POR patients. (M = methylated primer, UN = unmethylated primer). The 1 and 2 bands are for MSP positive control of UN methylated gDNA (10%). The 7 and 8 bands are for MSP positive control of UN methylated gDNA (20%). The 13 and 14 bands are for MSP positive control of fully methylated placental gDNA (100%). The 3, 4, 9 and10 bands are for HLA-G and the 5, 6, 11 and12 bands are for ICAM-1. The (3, 4) and (5, 6) bands are for POR patients with healthy lifestyle habits and (9, 10) and (11, 12) bands are for POR patients without healthy lifestyle habit. Columns 1, 3, 5, 7, 9, 11 and 13 belong to the patient sample with unmethylated primer and columns 2, 4, 6, 8, 10, 12 and 14 belong to the patient sample with methylated primer. Sample No. 1, 2, 7, 8, 13, and 14 bands are positive controls (DNA samples of fertile women). Sample No. 3, 4, 5, 6, 9, 10, 11, and 12 belong to different patient samples.

The DNA methylation status of *ICAM-1* and *HLA-G* genes in COCs samples was evaluated by the methylation-specific PCR (MSP). For MSP, we had to use specific primer pairs for both methylated and unmethylated promoter sequences. The primers were designed using the Meth primer and GeneRunner software. Each MSP reaction was performed in a total volume of 25 μL. One microliter of sodium bisulfite converted DNA was added into a 24 μL reaction mixture containing 0.5 U of hot start Gold Taq Polymerase (Cat NO: M5001, Promega, USA), 5 μL of the 10× PCR buffer, 2.0 μL of MgCl_2_ (50 mmol/L), 0.5 μL of dNTP (10 mmol/L; Fermentas, USA) and 1 μL of the corresponding forward and reverse primers (10 μmol/L dH2O up to final volume of 25 μL). Sodium bisulfite treated DNA was amplified in two separate MSP reactions, one with a set of primers specific for methylated, and the one for unmethylated *ICAM-1* and *HLA-G* promoters sequences (Table 1). For MSP positive control, we used fully methylated DNA (100%) and unmethylated DNA (0%), which the fully methylated DNA was made of MsssI methylase (NEB, Ipswich, MA, USA) using human placental genomic DNA (gDNA; Sigma Aldrich, USA), and the unmethylated DNA was made of human placental genomic DNA (gDNA; Sigma Aldrich, USA). For MSP method sensitivity, we used the fully methylated and unmethylated DNA (EpiTect Control DNA and Control DNA Set, Qiagen, Germany) with different serial dilutions: the ratio 0% to 100%, 5% to 95%, 10% to 90%, 20% to 80%, 35% to 65%, 50% to 50%, 75% to 25% and 100% to 0% unmethylated DNA was combined with methylated DNA, respectively. Thermo cycling conditions were as follows: 95 °C for 5 min followed by 40 cycles of 95 °C for 30 s, 63 °C for 60 s and 72 °C for 60 s, with a final extension of 72 °C for 4 min. MSP products for methylated and unmethylated *ICAM-1* and *HLA-G* promoters were run on 2% agarose gels containing 40 mMTris-acetate/1.0 mM EDTA (pH = 8) and were visualized by ethidium bromide staining (Fig. 2). Methylation genes pattern visualization was performed using the enhanced chemiluminescence detection system (UVITEC, UK). The intensity of each MS-PCR bands on the agarose gel was quantified using the ImageJ software version 1.50i (http://imagej.nih.gov/ij/; provided in the public domain by the National Institutes of Health, Bethesda, MD, USA).

### Data analysis

Relative gene expressions were calculated by the 2^−ΔΔCt^ method. The normalized ΔCt value of each sample was calculated using reference genes. In this study, categorical variables were presented as number (%) and continuous variables as mean ± SD. The independent t-test was used to assess the mean differences in demographic and clinical characteristics between the groups. Chi-square analysis was used for qualitative data. Univariate and backward multiple linear regression, including all variables, were used to evaluate the association between BPA concentration and *ICAM-1* and *HLA-G* genes, proteins, methylation and demographic and clinical variables. Statistical analyses were conducted using the IBM SPSS Statistics for Windows, Version 22.0 (IBM Crop., Armonk, NY, USA). All statistical tests were 2-tailed, and a P < 0.05 was considered statistically significant.

### Informed consent

All participants gave informed consent prior to inclusion in the study**.** All methods were carried out in accordance with relevant guidelines and regulations.

## Results

### Demographic data analysis

Table 2 presents the participants’ demographic and clinical characteristics. The number of oocytes was significantly higher in the healthy lifestyle habit group (Group1) than in the group without a healthy lifestyle habit (Group 2), (3.83 ± 0.99 vs. 3.27 ± 0.64, p = 0.004, respectively). The number of the MII retrieved oocyte was higher in Group 1 than in Group 2 significantly (2.83 ± 0.99 vs. 2.35 ± 0.66, p = 0.013, respectively). The clinical pregnancy rate (fetal heart detection by ultrasound) was significantly different between the two groups. Indeed, the clinical pregnancy rate was higher in the healthy lifestyle habit group than in Group 2 (1.80 ± 0.41 vs. 1.70 ± 0.46, 0.040). In the clinical variables of total embryo number and oocyte quality between two groups was significantly difference. Actually, the number of total embryo and oocyte quality was significantly higher in Group 1 than in Group 2 (oocyte quality: 3.51 ± 1.05 vs. 2.50 ± 0.93, p = 0.000; total embryo: 1.93 ± 026 vs. 1.30 ± 0.56, p = 0.000). In addition, a significant difference was observed in the AFC level between the two groups (p = 0.05). On the other hand, in the age, marriage duration, infertility duration, BMI, and AMH level of patients, there was no difference significantly.

**Table 2:** Clinical parameters for without healthy lifestyle habit and with healthy lifestyle habit of POR patients.

(n = 80)	Without healthy lifestyle habit	With healthy lifestyle habit	P-value
Age (year)	29.60 ± 3.77	29.66 ± 4.26	0.948
Marriage duration (year)	4.15 ± 0.89	4.39 ± 1.24	0.322
Infertility duration (year)	3.23 ± 0.80	3.59 ± 1.16	0.109
BMI	23.22 ± 1.27	24.31 ± 1.04	0.642
AMH	1.30 ± 0.22	1.39 ± 0.31	0.123
AFC	5.95 ± 1.04	6.49 ± 1.38	0.051
No. oocyte	3.27 ± 0.64	3.83 ± 0.99	0.004*
No. MII	2.35 ± 0.66	2.83 ± 0.99	0.013*
Oocyte quality	2.50 ± 0.93	3.51 ± 1.05	0.000*
Total embryo	1.30 ± 0.56	1.93 ± 026	0.000*
No. clinical pregnancy	1.80 ± 0.41	1.70 ± 0.46	0.040
Concentration of follicular fluid BPA	4.73 ± 2.23	1.56 ± 1.33	0.0001*
*ICAM1* gene (fold change)	1.14 ± 0.65	14.1 ± 4.52	0.000*
*HLA-G* gene (fold change)	1.17 ± 0.63	14.11 ± 4.52	0.000*
*ICAM1* DNA methylation (fold change)	1.20 ± 0.57	15.13 ± 4.28	0.000*
*HLA-G* DNA methylation (fold change)	1.25 ± 0.53	15.21 ± 4.33	0.000*
ICAM1 protein (fold change)	0.98 ± 0.07	1.22 ± 0.12	0.000*
HLA-G protein (fold change)	0.98 ± 0.07	1.22 ± 0.12	0.000*

Continuous variables are presented as mean ± SD.

AFC: antral follicular count AMH: anti Müllerian hormone, MII: Retrieved oocytes were classified into metaphase II, BPA concentration value is reported as ng/mL.

*P < 0.05 was considered significant.

After adjusting all variables (such as; the clinical outcome, gene expression data, and protein expression data), based on the multiple linear regression model results, the AMH level had a negatively significant association with the ICAM1 and HLA-G proteins expression (p = 0.018); for each unit increase in the AMH level, the expected oocyte quality was decreased by 0.14. The multiple linear regression model indicated that the number of MII was positively associated with the ICAM1 and HLA-G proteins expression (p = 0.021); for each unit increase in AMH, the expected ICAM1 and HLA-G proteins expression was increased by 0.04. This multivariate model revealed a negatively association with *ICAM1* and *HLA-G* genes in two groups studied. Although the ICAM1 and HLA-G proteins expression in women without a healthy lifestyle habit were decreased by nearly 13 times than in women with a healthy lifestyle habit (p = 0.000), the expected ICAM1 and HLA-G proteins expression were decreased by nearly 0.23 times in women without a healthy lifestyle habit than in women with a healthy lifestyle habit (p = 0.000, Table 4). The rest of the variables included in the univariate model (Table 3) were not significantly associated with oocyte quality in multiple models.

**Table 3:** Univariate regression analysis for factors associated with ICAM1and HLA-G genes, proteins and DNA methylation.

	*ICAM1* gene			*HLA-G* gene			ICAM1 protein			HLA-G protein			BPA concentration		
	B	SE	P value	B	SE	P value	B	SE	P value	B	SE	P value	B	SE	P value
Age	0.105	0.204	0.609	0.108	0.204	0.598	− 0.005	0.004	0.277	− 0.004	0.004	0.322	0.235	0.334	0.987
AMH	3.11	3.014	0.305	3.09	3.01	0.307	0.50	0.065	0.444	0.044	0.065	0.499	2.01	2.036	0.645
AFC	1.15	0.65	0.079	1.14	0.64	0.081	0.024	0.014	0.088	0.022	0.014	0.108	2.98	0.36	0.022
No .oocyte	1.99	0.903	0.030	1.98	0.901	0.031	0.058	0.019	0.003	0.056	0.019	0.004	2.09	0.453	0.012
No. MII	1.66	0.91	0.073	1.66	0.91	0.073	0.051	0.019	0.009	0.049	0.019	0.013	1.22	0.85	0.023
Oocyte quality	2.62	0.67	0.000	2.62	0.67	0.000	0.052	0.015	0.001	0.050	0.015	0.001	1.32	0.53	0.000
Total embryo	7.34	1.28	0.000	7.32	1.28	0.000	0.149	0.028	0.000	0.147	0.028	0.000	5.22	2.23	0.000
No. pregnancy	− 0.91	1.89	0.631	− 0.903	1.88	0.632	− 0.022	0.040	0.589	− 0.017	0.0 40	0.674	− 0.71	2.86	0.968
Group (without healthy lifestyle habits, with healthy lifestyle habit)	− 12.96	0.722	0.000	− 12.96	0.721	0.000	− 0.242	0.022	0.000	− 0.240	0.022	0.000	− 10.06	0.968	0.000

B: unstandardized coefficient; SE: standard error; AFC: antral follicular count; AMH: anti Müllerian hormone.

### ICAM1 and HLA-G mRNA, protein expressions, and DNA methylation pattern

**Figure 3:**
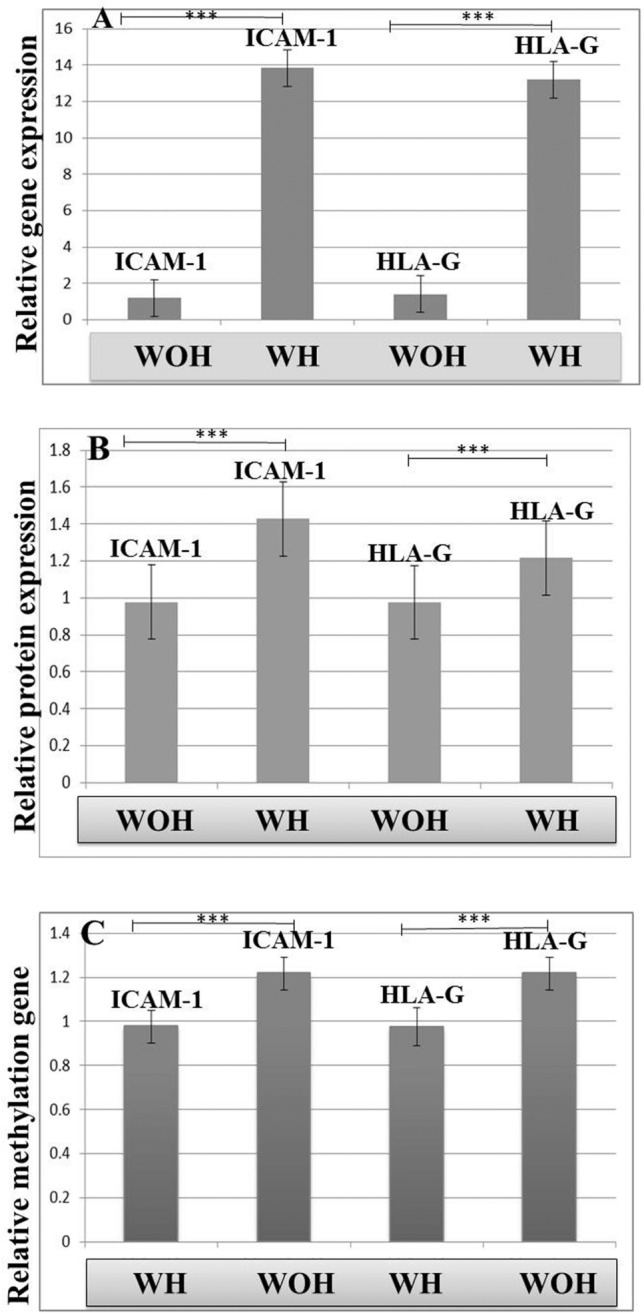
(**A**) Comparison of *ICAM-1* and *HLA-G* genes expression of q-PCR analysis. (**B**) *ICAM-1* and *HLA-G* proteins expression and, (**C**) *ICAM-1* and *HLA-G* DNA methylation status between without healthy lifestyle habit (WOH) and with healthy lifestyle habit (WH) of POR patients. *P < 0.05, **P < 0.01, ***P < 0.001 was considered significant. N = 40, (40 patient in each group was assessed).

The mean relative expression of *ICAM1* and *HLA-G* genes was significantly increased in Group 1 (p = 0.000) than in Group 2 (Fig. 3a).

For analysis of HLA-G and ICAM proteins, western blotting test was performed. The data showed ICAM1 and HLA-G proteins had expression in the cumulus cells of POR patients (Fig. 1). Analysis of protein bands intensity data was significantly increased in Group 1 (p = 0.000) than Group 2 (Fig. 3b).

The methylation pattern of *HLA-G* and *ICAM-1* genes was evaluated by MS-PCR (MSP, Fig. 2). Analysis of MSP bands showed that a notable increase in *ICAM1* and *HLA-G* DNA methylation in Group 2 compared to Group 1 (p = 0.000, Fig. 3c).

The mean of DNA methylation index for *HLA-G* and *ICAM-1* genes in the cumulus cells were significantly lower in Group 1 compared with Group 2, (0.13 ± 0.008, 0.15 ± 0.007, 0.25 ± 0.008 and 0.27 ± 0.009, p = 0.000, respectively, Fig. 3c).

### BPA concentration and the association with expressions and DNA methylation pattern

The mean concentration of BPA in follicular fluid was statistically significantly lower in Group 1 than in Group 2 (1.56 ± 1.33 ng/mL and 4.73 ± 2.23 ng/mL, P < 0.0001 respectively), (Table 2).

Based on the univariate analysis (Tables 3, 4), oocyte number (p = 0.03), oocyte quality, total embryo, and type of group (p = 0.000) in the groups studied (women with and without a healthy lifestylehabit) were significantly associated with BPA concentration, *ICAM1* and *HLA-G* gene expression and DNA methylation.

**Table 4:** Multivariate regression analysis for factors associated with *ICAM1*and *HLA-G* genes and proteins.

	*ICAM1* gene			*HLA-G* gene			icam1 protein			HLA-G protein			BPA concentration		
	B	SE	P value	B	SE	P value	B	SE	P value	B	SE	P value	B	SE	P value
AMH							− 0.148	0.061	0.018	− 0.149	0.062	0.018	− 0.122	0.073	0.026
No. MII							0.048	0.019	0.016	0.046	0.020	0.021	0.033	0.010	0.072
Group (without healthy lifestyles, with healthy lifestyle)	− 12.96	0.722	0.000	− 12.96	0.721	0.000	− 0.233	0.022	0.000	− 0.231	0.022	0.000	− 0.763	0.016	0.000

B: unstandardized coefficient; SE; standard error; AMH: anti Müllerian hormone.

Variables of oocyte number, oocyte quality, total embryo, MII number and type of group were significantly associated (Table 2) with ICAM1 and HLA-G proteins expression in the groups studied.

## Discussion

In the present study, our finding showed that concentration of BPA in follicular fluid of POR with healthy lifestyle was lower compared to the POR without healthy lifestyle habit, also, *ICAM-1*and *HLA-G* transcripts and proteins in POR with healthy lifestyle habit were upregulated compared to the POR without a healthy lifestyle habit. In addition, the *ICAM-1* and *HLA-G* DNA methylation status in the POR with a healthy lifestyle habit was hypomethylated compared to the POR without a healthy lifestyle habit. In fact, BPA can affect the transcript and proteins profile alterations in cumulus cells from POR patients. Furthermore, it may affect the methylation status in the cumulus cells of POR patients. Moreover, based on our data, the groups without a healthy lifestyle habit showed significantly lower numbers of good quality oocytes and clinical pregnancy rate compared to the group with a healthy lifestyle habit. As a result, BPA can affect the ovulation, oocyte maturation and clinical pregnancy rate in POR patients. Oocyte quality is one of the major limiting factors for the success of the ART cycle in POR patients^18^. Oocyte maturation is achieved during folliculogenesis. Folliculogenesis is the maturation of the ovarian follicle. Folliculogenesis needs communication between the oocyte and surrounding somatic cells^19^. One of the kinds of somatic cells is cumulus cells. Cumulus cells play a potential role in achieving oocyte development^20^. Based on a recent study, some genes are expressed in the cumulus cells that can be considered genetic markers for the association between oocyte and embryo quality. Probably, developmental signals from cumulus cells are transferred to the oocyte via gap junctions and pathways, which can affect the oocyte maturation^21^. The present study aimed to determine the novel essential marker to evaluate the oocyte quality and clinical pregnancy rate in the ART treatment of POR patients, and our data showed that lifestyle habit might be considered an essential marker for oocyte maturation increasing the clinical pregnancy rate. One of the consequent complications of POR is oocyte maturation disorder^5^. Nowadays, overuse of plastic objects to store hot food and hot drinks has caused numerous health problems in humans like reproduction disorders, including POR^22^. In fact, plastic objects made of polycarbonate and polycarbonates contain BPA^7^. According to the recent study, BPA may affect the infertility related gene expression that is closely associated with reproductive, but this association interaction mechanism is unclear. Owing to heat, acidic or alkaline substances, polycarbonate will break and BPA can attach to food and drink, and it can enter the body^6^. BPA can act like sex hormones, so that it can disrupt the endocrine system and hormonal signaling pathways. BPA in small amounts may interfere in the reproduction related gene expression, and it can interfere in the function of ovary, uterus and other reproductive organs^22^. Pednekar et al.^11^ reported that infertile women showed significantly higher plasma concentrations of BPA. As a result, lifestyle habit plays a vital role in the regulating of ovarian response, oocyte maturation, reproductive process and genes expression. In this study, we evaluated the expression profile for *ICAM-1* and *HLA-G* genes in the cumulus cells of infertile women with poor response to ovarian stimulation (POR) based on their healthy lifestyle habit. The probable effect of plastic objects usage was observed on the genes, proteins, DNA methylation pattern, oocyte quality and clinical pregnancy rate in these women. According to other studies, altered gene expression of CCs may play a role in women with POR pathogenesis^23,24^. Therefore, assessment of genes expression is valuable to understand the etiology of infertile women with POR patients, since oocyte maturation dysfunction is one of the disorders in POR patients^11^. Recently, it has been shown that alternation of *ICAM1* and *HLA-G* genes can affect the quality and quantity of oocytes^25^. Our findings revealed that a major contributor to oocyte maturity disorder and infertility in the POR patients without a healthy lifestyle habit can be a reduction in *ICAM1* and *HLA-G* transcript and proteins expression as well as hypermethylation of DNA in *ICAM1* and *HLA-G*. According to recent studies, comparison of POR patients to healthy, normal ovulatory fertile women with a history of male infertility history has shown differential expression of *ICAM-1* genes^26^.

The *ICAM-1* gene expression pattern indicated that it is important in oocyte maturity and grading. It appeared that dysregulation of *ICAM-1* expression could be associated with defective oocyte maturation and grading^27^.The studies demonstrated that *ICAM-1* was a probable key effector of the oocyte maturation. Although the sICAM-1 release was very high in immature oocytes, it was decreased in mature oocytes. They regarded sICAM-1 as a biochemical marker for oocyte maturation and grading^11^. The ICAM-1 appears to play a critical role in oocyte development, while ICAM-1 levels play the role of a predictive marker in oocyte maturation and quality. Borgtti and Rizzo^25^ proposed sICAM-1 as a biochemical marker for oocyte maturation and grading. Their findings supported a significant correlation between *ICAM-1* gene and protein expression and oocyte maturation. A recent study has shown that *ICAM-1* is expressed on leukocytes, epithelial and endothelial cells^11^. Furthermore, HLA-G plays a vital role in the oocyte maturation and preimplantation of embryos. Jurisicova et al. detected HLA-G mRNA expression by preimplantation human embryos^28^. They demonstrated the presence of HLA-G mRNA and protein in the blastocysts tested^13^. Yao et al.^29^ revealed the expression of the HLA-G protein by human preimplantation embryos with an increasing ratio of positive embryos with developmental stage. Kaneko^28^ indicated that the low expression of HLA-G in cumulus cells was associated with lower oocyte quality, poor fertilization, and reduced embryo development^29^. Rooij van reported the low level of HLA-G in the cumulus cells of POR patients^12^. The gene expression alternation in the cumulus cells of women with POR after dehydroepiandrosterone (DHEA) supplementation can affect the oocyte maturation^23^. According to recent study, oocyte quality decreases with increasing age^24^. Our findings indicated that age was directly associated with oocyte quality and aging. Furthermore, another study showed that younger POR patients’ quality of oocyte and grading were better than older patients’^30^. Our study also highlighted the role of age as one of the components in oocyte maturation. The present study emphasized the association of lifestyle habit with the *ICAM1* and *HLA-G* genes expression alteration and oocyte quality as an opinion for the deeper understanding of the etiology of infertile women with POR patients.

## Conclusion

The current study was the first single center lifestyle program of POR patients in Iran. We found that alterations of *ICAM1* and *HLA-G* in POR appeared to be related to BPA concentration. As a result nutrition and lifestyle habit are affecting in genes function. Indeed, the nutrition and lifestyle habit may be effective on the methylation pattern, expression profile and proteins level in cumulus cells from POR. Moreover, nutrition and lifestyle habit may act as an essential marker for ovulation and the oocyte maturation process.

## Limitation

Sample collection was the main limitation in our experiment as well as severe limitation of using laboratory facilities because of the COVID-19 pandemic situation.

## Supplementary Information

Supplementary Information.

## Data Availability

They are available on the request.

## Abbreviations

POR: Poor ovarian response
AMH: Anti Müllerian hormone
AFC: Antra follicle count
BPA: BisphenolA
ECM: Extracellular matrix
HLA-G: Human leukocyte antigen-G
CCs: Cumulus cells
GnRH: Gonadotropin releasing hormone
ICSI: Intracytoplasmic sperm injection
COS: Controlled of ovarian stimulation
SC: Subcutaneous
RFSH: Recombinant follicle stimulating hormone
E2: Estradiol
HCG: Human chorionic gonadotropin
IM: Intramuscular
CoCs: Cumulus-oocyte complexes
PBS: Phosphate-buffered saline
MII: Metaphase II gametes
MSP: Methylation-specific polymerase chain reaction
DHEA: Dehydroepiandrosterone

## References

[1] Ferraretti A-P, Marca A, Fauser B-C, Tarlatzis B, Nargund G, Gianaroli L (2011). ESHRE working group on Poor Ovarian Response. Definition ESHRE consensus on the definition of ‘poor response’ to ovarian stimulation forin vitro fertilization: The Bologna criteria. Hum. Reprod..

[2] Nagels HE (2015). Androgens (dehydroepiandrosterone or testosterone) for women undergoing assisted reproduction. Cochrane Database Syst. Rev..

[3] Pandian Z, McTavish A-R, Aucott L, Hamilton P-L, Bhattacharya S (2010). View authors' declarations of interest Interventions for ‘poor responders’ to controlled ovarian hyper stimulation (COH) in in-vitro fertilisation (IVF). Cochrane Database Syst. Rev..

[4] Humaidan P, Alviggi C, Fischer R, Esteves S-C (2016). The novel POSEIDON stratification of ‘Low prognosis patients in Assisted Reproductive Technology’ and its proposed marker of successful outcome. F1000 Research..

[5] Patrizio P, Vaiarelli A, Setti P-E-L, Tobler K, Shoham G, Leong M, Shooham Z (2015). How to define, diagnose and treat poor responders? Responses from a worldwide survey of IVF clinics. Reprod. Biomed. Online..

[6] Xiaona H, Dan C-H, Yonghua H-E, Wenting Z, Wei Z, Jun Z (2015). Bisphenol-A and female infertility: A possible role of gene-environment interactions. Int. J. Environ. Res. Public Health..

[7] Liu F, Liu W-N, Zhao Q-X, Guo W, Zhuang R, Jia X-D (2013). Study on environmental and psychological risk factors for female infertility. Chin. J. Ind. Hyg. Occup. Dis..

[8] Nishikawa M, Iwano H, Yanagisawa R (2010). Placental transfer of conjugated bisphenol A and subsequent reactivation in the rat fetus. Environ. Health Perspect..

[9] Dang V-H, Choi K-C, Jeung E-B (2007). Tetra bromo diphenyl ether (BDE 47) evokes estrogenicity and calbindin-D9k expression through an estrogen receptor-mediated pathway in the uterus of immature rats. Toxicol. Sci..

[10] Choi K-C, Jeung E-B (2003). The biomarker and endocrine disruptors in mammals. J. Reprod. Dev..

[11] Borgatti M, Rizzo R, Dal Canto MB, Fumagalli D, Renzini MM, Fadini R, Stignani M, Baricordi OR, Gambari R (2008). Release of sICAM-1 in oocytes and in vitro fertilized human embryos. PLoS ONE.

[12] Rebmann V, Switala M, Eueb I, Schwah E, Merzenich M, Wilde H-G (2007). Rapid evaluation of soluble HLA-G levels in supernatants of in vitrofertilized embryos. Hum. Immunol..

[13] Uzzi B, Rizzo R, Ivo N, Melchiorri L, Scarselli B, Bencini E, Menicucci A, Baricordi O-R (2002). HLA-G expression in early embryos is a fundamental prerequisite for the obtainment of pregnancy. Eur. J. Immunol..

[14] Oci I, Fuzzi B, Rizzo R, Melchiorri L, Criscuoli L, Dabizzi S, Biagiotti R, Pellegrini S, Menicucci A, Baricordi O-R (2005). Embryonic soluble HLA-G as a marker of developmental potential in embryos. Hum. Reprod..

[15] Wissing M-L, Sonne S-B, Westergaard D, Nguyen K-H, Belling K, Høst T-H, Mikkelsen A-L (2014). The transcriptome of corona radiata cells from individual MII oocytes that after ICSI developed to embryos selected for transfer: PCOS women compared to healthy women. J. Ovarian Res..

[16] Einstein A, Podolsky B, Rosen N (2009). Impact of insulin resistance on the developmental potential of immature oocytes retrieved from human chorionic gonadotropin primed women with polycystic ovary syndrome undergoing in vitro maturation. Fertil. Steril..

[17] Rahimizadeh P, Rezaei Topraggaleh T, Nasr-Esfahani MH, Ziarati N, Mirshahvaladi SH, Esmaeili V, Seifi S (2020). The alteration of PLCζ protein expression in unexplained infertile and asthenoteratozoospermic patients: A potential effect on sperm fertilization ability. Mol. Reprod. Dev..

[18] Kim K, Park Y, Im G (2013). Changes in the epigenetic status of the SOX-9 promoterin human osteoarthritic cartilage. JBMR..

[19] Knight PG, Glister C (2003). Local roles of TGF-beta superfamily members in the control of ovarian follicle development. Anim. Reprod. Sci..

[20] Cecconi S, Ciccare C, Barberi M, Macchiare G, Caniparic R (2004). Granulosa cell-oocyte interactions. Eur. J. Obstet. Gynecol. Reprod. Biol..

[21] Salehi E, Aflatoonian R, Moeini A, Yamini N, Asadi E, Khosravizadeh Z, Tarzjani M-D, Naghibiharat Z, Abolhassani F (2017). Apoptotic biomarkers in cumulus cells in relation to embryo quality in polycystic ovary syndrome. Gynecol. Obstet..

[22] Ziv-Gal, A. & Flaws, J.-A. Evidence for bisphenol A-induced female infertility. *Fertil. Steril.* **106**(4), 827–856 (2016–2017). 10.1016/j.fertnstert.2016.06.027 PMC5026908 27417731

[23] Tsuia K, Lin L, Horng H-C, Shian C-B, Chenga J-T, Wang P-H (2014). Gene expression of cumulus cells in women with poor ovarian response after dehydroepiandrosterone supplementation. Taiwan. J. Obstet. Gynecol..

[24] Van Rooij IAJ, Bancsi F-J-M-L, Broekmans F, Looman C-W-N, Habbema D, Velde E-R (2003). Women older than 40 years of age and those with elevated follicle-stimulating hormone levels differ in poor response rate and embryo quality in in vitro fertilization. Fertil. Steril..

[25] Thomson A, Greer M-R, Young A, Boswell F, Telfer J-F, Cameron I-T, Norman J-E, Campbell S (1999). Expression of intercellular adhesion molecules ICAM-1 and ICAM-2 in human endometrium: Regulation by interferon-g. Mol. Hum. Reprod..

[26] Oci I, Fuzzi B, Rizzo R, Melchiorri L, Criscuoli L, Dabizzi S, Biagiotti R, Pellegrini S, Menicucci A, Baricordi O-R (2005). Embryonics oluble HLA-G as a marker of developmental potential in embryos. Hum. Reprod..

[27] Opiela, J., Romanek, J., Lipiński, D. & Smorąg, Z. Effect of hyaluronan on developmental competence and quality of oocytes and obtained blastocysts from in vitro maturation of bovine oocytes. BioMed Res. Int. 519189 (2014). 10.1155/2014/519189 PMC3945031 24689043

[28] Jurisicova A, Casper R-F, MacLusky N-J, Librach CL (1996). Embryonic human leukocyte antigen-G expression: Possible implications for human preimplantation development. Fertil. Steril..

[29] Yao YQ, Barlow D-H, Sargent J-L (2005). Differential expression of alternatively spliced transcripts of HLA-G in human preimplantation embryos and inner cell masses. J. Immunol..

[30] Perna L, Zhang Y, Mons U, Holleczek B, Saum K-U, Brenner H (2016). Epigenetic age acceleration predicts cancer, cardiovascular, and all-cause mortality in a German case cohort. Clin. Epigenet..

