# Supplementary material for: Effect of bisphenol A on alterations of ICAM-1 and HLA-G genes expression and DNA methylation profiles in cumulus cells of infertile women with poor response to ovarian stimulation
Source: Sci Rep. 2021 May 5;11:9595. doi: 10.1038/s41598-021-87175-1 (PMC8099902; doi:10.1038/s41598-021-87175-1)

Effect of Bisphenol A on alterations of *ICAM-1* and *HLA-G* genes Expression and DNA methylation profiles in Cumulus Cells of Infertile Women with Poor Response to Ovarian Stimulation

Somayeh Aftabsavad[1], Zahra Noormohammadi[1]*, Ashraf Moini[2,3,4], Morteza Karimipoor[5].

1. Department of Biology, Science, and Research Branch, Islamic Azad University, Tehran, Iran
2. Department of Endocrinology and Female Infertility, Reproductive Biomedicine Research Center, Royan Institute for Reproductive Biomedicine, ACECR, Tehran, Iran
3. Breast Disease Research Center (BDRC), Tehran University Of Medical Science, Tehran,Iran.
4. Department of Obstetrics and Gynecology, Arash Women's Hospital, Tehran University of Medical Sciences, Tehran, Iran
5. Department of Molecular Medicine, Biotechnology Research Center, Pasteur Institute of Iran, Tehran, Iran

Original Figures

Fig1a

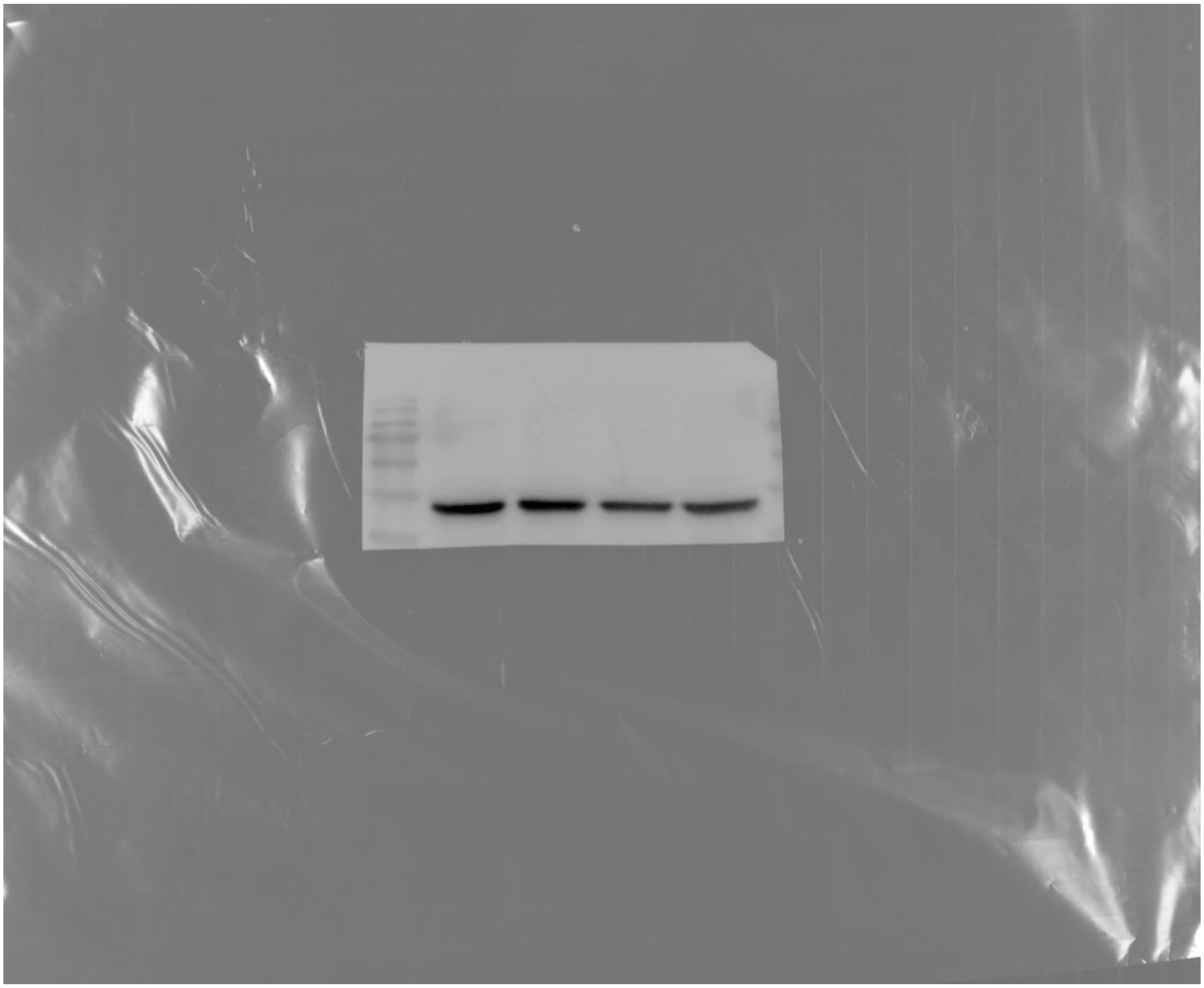

Fig1b

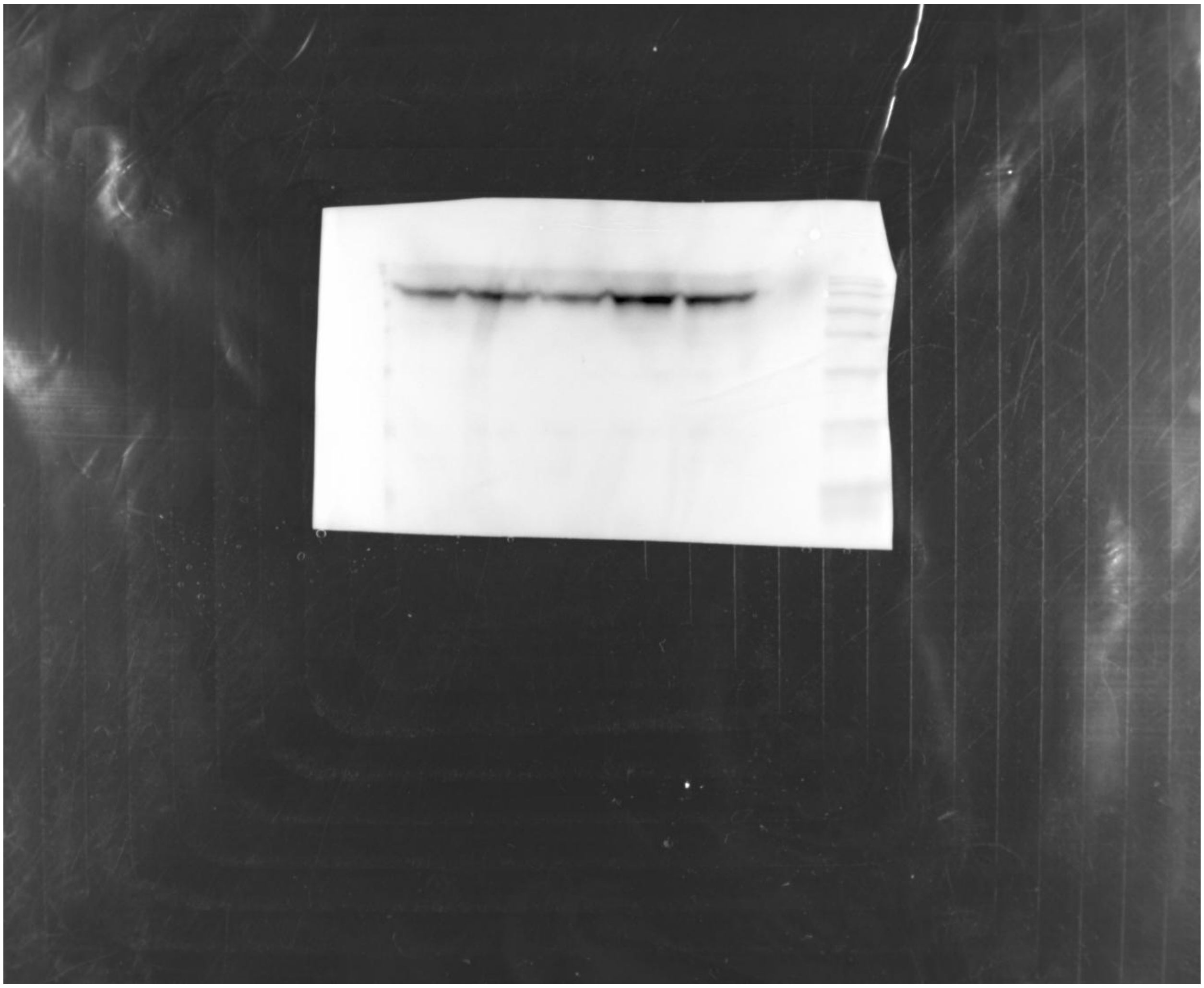

Fig1c

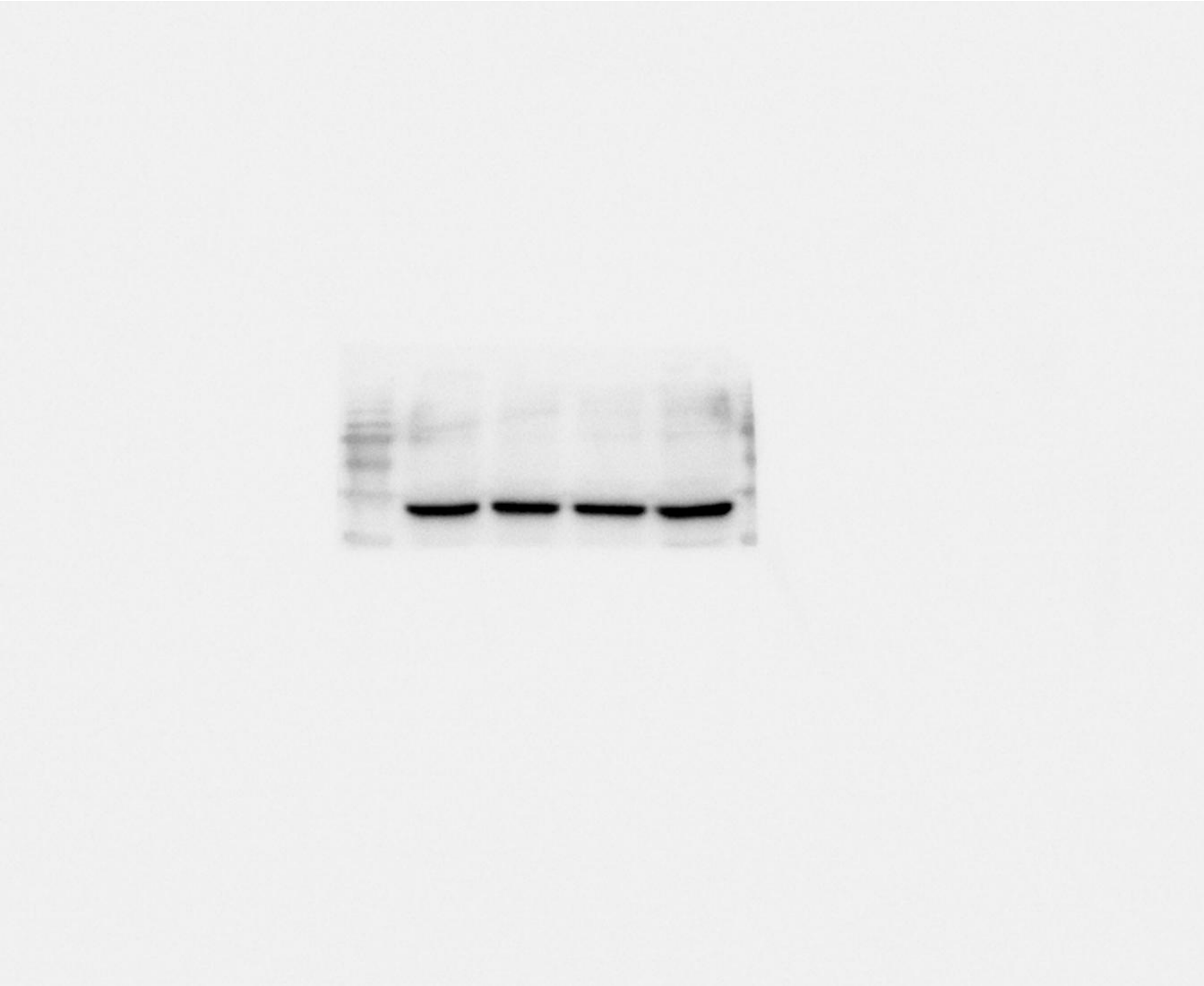

Supplement: Supplementary file 1 — Supplementary Information. [file 41598_2021_87175_MOESM1_ESM.pdf]
